# Diagnostic Performance of Vascular Permeability and Texture Parameters for Evaluating the Response to Neoadjuvant Chemoradiotherapy in Patients With Esophageal Squamous Cell Carcinoma

**DOI:** 10.3389/fonc.2021.604480

**Published:** 2021-05-18

**Authors:** Wenbing Ji, Jian Wang, Rongzhen Zhou, Minke Wang, Weizhen Wang, Peipei Pang, Min Kong, Chao Zhou

**Affiliations:** ^1^ Department of Radiology, Taizhou Hospital of Zhejiang Province, Taizhou, China; ^2^ Advanced Application Team, GE Healthcare, Shanghai, China; ^3^ Department of Thoracic Surgery, Taizhou Hospital of Zhejiang Province, Taizhou, China; ^4^ Department of Radiotherapy, Taizhou Hospital of Zhejiang Province, Taizhou, China

**Keywords:** neoadjuvant chemoradiotherapy, esophageal squamous cell carcinoma, dynamic contrast-enhanced magnetic resonance imaging, vascular permeability, texture parameter

## Abstract

**Background:**

Esophageal squamous cell carcinoma (ESCC) is an aggressive type of cancer, associated with poor prognosis. The development of an accurate and non-invasive method to evaluate the pathologic response of patients with ESCC to chemoradiotherapy remains a critical issue. Therefore, the aim of this study was to assess the importance of vascular permeability and texture parameters in predicting the response to neoadjuvant chemoradiotherapy (NACRT) in patients with ESCC.

**Methods:**

This prospective analysis included patients with T1–T2 stage of ESCC, without either lymphatic or metastasis, and distant metastasis. All patients underwent surgery having received two rounds of NACRT. All patients underwent dynamic contrast-enhanced magnetic resonance imaging (DCE-MRI) twice, i.e., before the first NACRT and after the second NACRT. Patients were assessed for treatment response at 30 days after the second NACRT. Patients were divided into the complete response (CR) and partial response (PR) groups based on their responses to NACRT. Vascular permeability and texture parameters were extracted from the DCE-MRI scans. After assessing the diagnostic performance of individual parameters, a combined model with vascular permeability and texture parameters was generated to predict the response to NACRT.

**Results:**

In this study, the CR and PR groups included 16 patients each. The volume transfer constant (Ktrans), extracellular extravascular volume fraction (ve), and entropy values, as well as changes to each of these parameters, extracted from the second DCE-MRI scans, showed significant differences between the CR and PR groups. The area under the curve (AUC) of Ktrans, ve, and entropy values showed good diagnostic ability (0.813, 0.789, and 0.707, respectively). A logistic regression model combining Ktrans, ve, and entropy had significant diagnostic ability (AUC=0.977).

**Conclusions:**

The use of a combined model with vascular permeability and texture parameters can improve post-NACRT prognostication in patients with ESCC.

## Introduction

Esophageal cancer (EC) ranks seventh in cancer incidence and sixth in mortality rate worldwide (1). EC has a poor prognosis and an aggressive phenotype, specifically in the advanced stages (2). In China, EC was the fourth leading cause of cancer-related mortality in 2015 (3). Pathological results showed that 95.5% of patients had esophageal squamous cell carcinoma (ESCC) (4). The standard therapy for ESCC includes surgery, radiotherapy, and chemotherapy (4). Neoadjuvant chemoradiotherapy (NACRT) has been recommended by the National Comprehensive Cancer Network for locally advanced ESCC or unresectable ESCC (2). In Western countries, clinical analysis has shown that patients with squamous cell carcinoma had better outcomes than those with adenocarcinoma (5). Patients with ESCC showed an improved pathologic complete response after NACRT (5). Pathologic complete response is considered to be one of the most important prognostic factors in ESCC (6–8) with respect to overall survival and disease-free survival. The prediction of the pathologic response before treatment could be useful in the selection of treatment.

Since the esophagus is located in the thoracic body cavity, the evaluation of the pathologic response is based on traditional imaging techniques, such as magnetic resonance imaging (MRI), computed tomography (CT), and esophagoscopy (9). These techniques qualitatively assess a pathologic response without a quantitative evaluation. Therefore, establishing a rapid, accurate, and non-invasive method to evaluate the pathologic response to NACRT remains a challenge. In recent years, dynamic contrast-enhanced (DCE) MRI scanning has been widely used in clinical trials to assess the changes in vascular permeability in various diseases (10) and to evaluate early responses in EC to chemoradiotherapy (11). With the development of image post-processing techniques, texture analysis has become a widely used method in clinical trials to evaluate tumor progression (12). However, developing a non-invasive method to evaluate pathologic responses to NACRT remains a challenge.

Therefore, the aim of this prospective study was to develop a non-invasive quantitative method using DCE-MRI scanning and texture analysis to assess the response of patients to NACRT and explore whether it could be used in post-NACRT prognostication of patients with ESCC.

## Materials and Methods

### Patients

Between July 2016 and June 2018, patients presenting at the Taizhou hospital of the Zhejiang province with histologically confirmed ESCC (i.e., clinical stage T1-T2, N0, M0, according to the TNM staging system of the American Joint Committee on Cancer) were eligible for the present study (13). All patients were evaluated using standard laboratory tests, esophagogastroduodenoscopies with endoscopic ultrasound, biopsies, CT scans, and MRI scans. The inclusion criteria were: 1) no prior anti-cancer therapy; 2) anticipated survival of > 6 months; 3) age 18 to 70 years; 4) absolute white blood cells count of ≥4.0×10^9^/L, neutrophil count of ≥1.5×10^9^/L, hemoglobin level of 90 g/L, and normal liver and kidney function; and 5) Karnofsky performance status score of ≥ 90. The exclusion criteria were: 1) diagnosed or suspected allergy to cisplatin or vinorelbine; 2) presence of concomitant hemorrhagic disease; 3) pregnancy or lactation; 4) any prior surgery and gastric conduit failure after esophagectomy; 5) concomitant peripheral neuropathy, with a common toxicity criteria of ≥ 2; and 6) any prior malignancy other than esophageal carcinoma, such as carcinoma *in situ* of the cervix, nonmelanoma skin cancer, or cured early-stage prostate cancer; 7) only 1 cycle completed of NACRT before surgery.

This study was approved by our institutional review board (NCT02188615) and informed consent was obtained from all patients included in the analysis.

### MRI Scanning

All patients underwent the first MRI scan before their first round of NACRT. The first MRI scan was scheduled 1–2 weeks before the administration of NACRT. All patients underwent the second MRI scan 1 week after the second round of NACRT.

### Patient Positioning, Coil Selection, and Examination

MRI scanning was performed using the Discovery MR750 HD 3.0T scanner (GE Healthcare, USA) with 8-channel abdominal coil for all patients. Before each MRI examination, each patient was guided through breathing exercises and provided with 200 mL of drinking water to remove esophageal residues. During the MRI examination, the patient was instructed to remain calm and refrain from swallowing. The MRI examination protocol included pre-contrast MRI and DCE-MRI scans. The pre-contrast MRI showed signals on 2D-T1 weighted images (T1WI), i.e., without contrast enhancement and the parameter of scanning was thus the same as that of DCE-MRI (2 periods), and 2D-T2WI (repetition time [TR]/echo time [TE] =3500–4000/80–95 ms, field-of-view=380 mm × 280 mm, acquisition matrix=352 ×352, slice thickness=5 mm, number of slices=30). The protocol for DCE-MRI scanning was as follows: 6 s per period over a total of 40 periods with the scanning time of 240 s (TR/TE=3.9/1.4 ms, flip angle=12°, field-of-view=380 mm×280 mm, acquisition matrix=320 ×224, slice thickness=5 mm, number of slices=30). After two-phase scanning, Gd-DTPA-BMA (Omniscan, GE Healthcare, Little Chalfont, UK) was injected with an automatic double tube high-pressure injector at a rate of 2 mL/s (0.1 mmol/kg of body weight). Subsequently, 20 mL saline was injected to flush the tube.

### NACRT

All patients were administered chemotherapy and radiotherapy at our hospital. In the preoperative radiotherapy regimen, the gross tumor volume included the primary esophageal tumor and metastatic lymph nodes; the clinical target volume (CTV) included the subclinical lesion (normal esophagus of 3 cm above and below esophageal tumor), and the corresponding para-esophageal lymphatic drainage area; the planned target volume included CTV plus a margin of 8 mm. A total dose of 25.0 to 30.0 Gy was administered in 10 fractions of 2.0 Gy/day, 5 times per week. The dose limit to the esophagus was of <15% of the volume. The cocurrent radiotherapy regimen comprised of cisplatin 25 mg/(m^2^/5 d) for 3 weeks. All patients received two cycles of NACRT; the total radiation dose was 50.0 to 60.0 Gy, and the total cisplatin dose was 150 mg/m^2^.

### Treatment Response

Thirty days after the second course of NACRT, all patients underwent surgery. Pathologic response was evaluated after surgery by two physicians, each with 10 to 15 years of experience in diagnostic histopathology. Pathological response to NACRT was classified into five grades: grade 1, the absence of residual cancer and fibrosis; grade 2, the presence of residual cancer cells scattered throughout fibrosis; grade 3, the presence of fibrosis and tumor cells, with fibrosis predominant; grade 4, the presence of fibrosis and tumor cells, with tumor cells predominant; finally, grade 5, the absence of regressive changes. We defined grades 1 and 2 as complete response (CR) and grades 3 to 5 as partial response (PR) (14). According to the evaluation results, patients were divided into the CR and PR groups and 16 patients were included in each group.

### Image Analysis

The artifacts related to breathing motion on DCE-MRI scans were corrected using a non-rigid calibration method in OmniKinetics software (GE Healthcare, Shanghai, China) (15). After importing the multi-flip angle sequence image into the software, the T10 value was calculated based on the MRI signal in the multi-flip angle image. The abdominal aorta was selected based on the DCE-MRI multi-phase dynamic image to obtain the arterial input function (AIF). During dynamic scanning, we calculated T_1t_, using the following equation (16, 17):

Equation 1, $$S(t)=S(0)\frac{1-e^{-\frac{T_{R}}{T_{1(0)}}}\cos\;\alpha }{1-e^{-\frac{T_{R}}{T_{1(0)}}}}\cdot \frac{1-e^{-\frac{T_{R}}{T_{1(t)}}}}{1-e^{-\frac{T_{R}}{T_{1(t)}}}\cos\;\alpha }$$

where S(t) is the MRI signal intensity over time during DCE-MRI, S_0_ is the pre-contrast signal intensity, TR is the repetition time, T_1(t)_ is the value of T_1_ over time after contrast injection, and α is the flip angle value.

Then, we calculated Ct using the following equation:

Equation 2, $$\frac{1}{T_{1(t)}}=\frac{1}{\gamma T_{1(0)}}\frac{s(t)}{S(0)}$$

Equation 3 $$C(t)=\frac{1}{\gamma }\left(\frac{1}{T_{1(t)}}-\frac{1}{T_{1(0)}}\right)=\frac{1}{\gamma T_{1(t)}}\frac{S(t)-S(0)}{S(0)}$$

C_t_ is the concentration of contrast in ROI over time during DCE-MRI where r1 is the relaxivity value of contrast.

The AIF, which is a time-concentration curve of the abdominal aorta, was obtained, using the above equation. The extended Tofts linear dual-chamber model based on the time resolution of the sequence of DCE-MRI was selected to generate the vascular permeability parameters: volume transfer constant (Ktrans), rate constant (Kep), extracellular extravascular volume fraction (ve), and plasma volume fraction (vp) (4). Physicians who performed the diagnosis analyzed lesion segmentation, extracted vascular permeability parameters, and texture parameters: entropy (**Supplementary Material 1**, **Supplementary Equation 2**), energy (**Supplementary Material 1**, **Supplementary Equation 1**), inertia (**Supplementary Material 1**, **Supplementary Equation 3**), correlation (**Supplementary Material 1**, **Supplementary Equation 4**), clustering (**Supplementary Material 1**, **Supplementary Equations 5** and **6**), and the inverse difference moment of the three-dimensional (3D) lesions) (**Supplementary Material 1**, **Supplementary Equation 7**). These texture parameters were calculated after vascular permeability parameters with OmniKinetics software; methodological details are presented in **Supplementary Material 1** (5).

### 3D Tumor Segmentation

The ROI of the 3D tumor was segmented by two physicians, each with 10 to 15 years of experience in diagnostic radiology, who were blinded to the pathology results. The physicians carefully segmented the entire tumor, according to the T1WI and the DCE-MRI scans, by manually sketching the outline of the entire tumor. During tumor segmentation, the physicians excluded the bleeding area, necrotic area, cyst, edema, and large vessels. After tumor segmentation, the vascular permeability was calculated using the OmniKinetics software (GE Healthcare, China).

### Statistical Analyses

Statistical analyses were performed using the packages of glmnet, pROC, and rms in the R software (version 3.4.0). Statistical significance for the two-sided tests was set at P-values of <0.05. The Mann–Whitney U test was used to analyze the changes in the vascular permeability and texture parameters during NACRT between the CR and PR groups. Parameters with P-values of <0.05 in Mann-Whitney U test were included in Spearman correlation analysis to reduce model overfittingKep. Features would be excluded at correlation coefficient values of ≥0.6. Logistic regression was used to build a model evaluating the CR rate after feature dimension reduction. Receiver operating characteristic (ROC) curve analysis was used to calculate the area under the curve.

## Results

### Comparison of Vascular Permeability Parameters Between the CR and PR Groups

**Figure 1:**
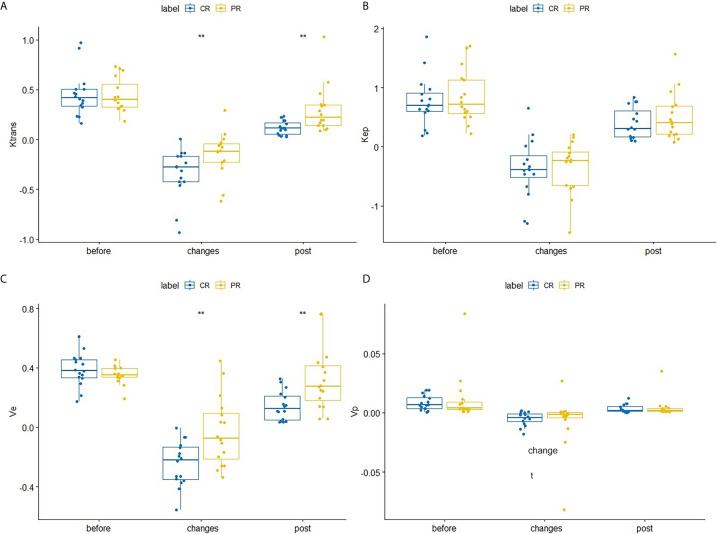
Differences in Ktrans **(A)**, Kep **(B)**, ve **(C)**, and vpV_p_ **(D)** between the CR and PR groups at the first NACRT, change, and the second NACRT, while the median value of relative vascular permeability parameter levels is displayed as a line within each box. ** means significant difference.

After the second NACRT, Ktrans (p=0.002) and ve (p = 0.002) showed significant differences between the CR and PR groups (**Table 1** and **Figures 1A, C**), the change in these parameters were similar for both groups (change =2^nd^ NACRT–1^st^ NACRT) (**Table 1** and **Figures 1A, C**) (Ktrans, p=0.008, ve, p=0.005). Even though the values of these parameters in the CR group were lower than in the PR group, differences between groups were not significant after the second NACRT and change in parameters (**Table 1** and **Figures 1B, D**). None of the vascular permeability parameters examined before the first NACRT showed significant differences between the CR and PR groups (p>0.05, **Table 1** and **Figure 1**).

**Table 1:** Differences between CR and PR for vascular permeability parameters.

Quantitative parameters	CR (n=16)*x̅* ± *S*	PR (n=16)*x̅* ± *S*	P-value
Ktrans (min^−1^)			
1^st^ NACRT	0.454 ± 0.213	0.444 ± 0.162	0.867
Change	−0.337 ± 0.235	−0.146 ± 0.212	0.008^**^
2^nd^ NACRT	0.117 ± 0.065	0.299 ± 0.231	0.002^**^
Kep(min^−1^)			
1^st^ NACRT	0.771 ± 0.412	0.849 ± 0.432	0.838
Change	−0.376 ± 0.482	−0.338 ± 0.423	0.515
2^nd^ NACRT	0.395 ± 0.254	0.512 ± 0.387	0.491
ve			
1^st^ NACRT	0.388 ± 0.107	0.352 ± 0.057	0.239
Change	−0.258 ± 0.147	−0.066 ± 0.234	0.005^**^
2^nd^ NACRT	0.130 ± 0.094	0.322 ± 0.204	0.002^**^
vp			
1^st^ NACRT	0.008 ± 0.006	0.011 ± 0.020	0.402
Change	−0.005 ± 0.005	−0.007 ± 0.217	0.468
2^nd^ NACRT	0.003 ± 0.003	0.004 ± 0.008	0.926

x̅ ± S, mean ± standard deviation. ** means significant difference.

### Comparison of Texture Parameters Between the CR and PR Groups

**Figure 2:**
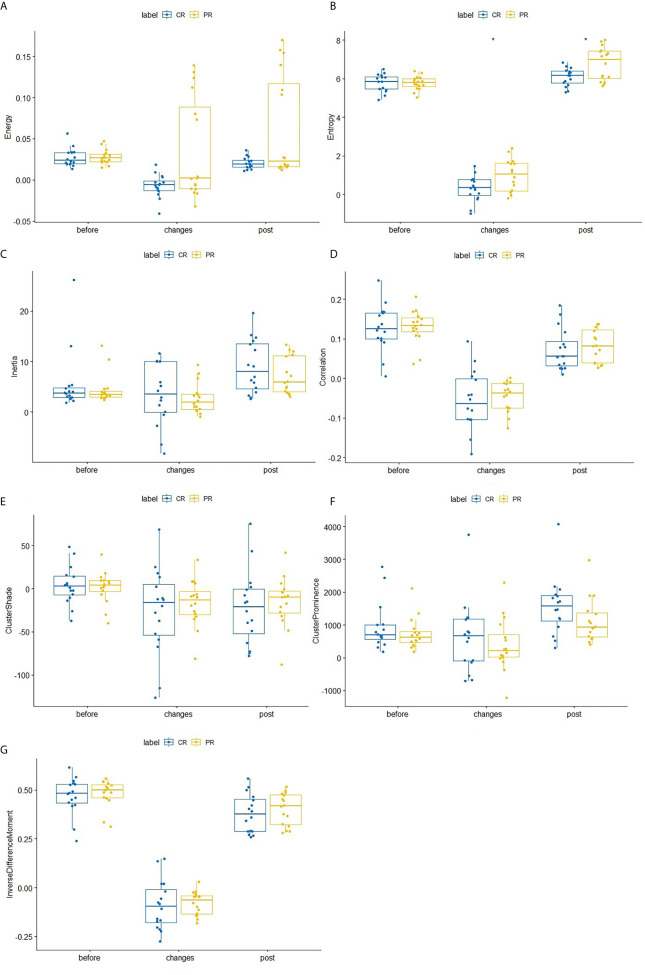
Differences in texture parameters between the CR and PR groups at the first NACRT, change, and the second NACRT, while the median value of relative texture parameter levels is displayed as a line within each box. **(A–G)** Energy, Entropy, Inertia, Correlation, Clustershade, ClusterProminence, InverseDifferenceMoment. AUC, area under the receiver operating characteristic curve; CI, confidence interval. * means significant difference.

After the second NACRT, significant differences in entropy values were observed between the CR and PR groups (p=0.047), change in entropy(p=0.032). No significant differences in texture parameters were observed in either group before either round of the NACRT; however, there were differences in entropy levels during the second NACRT and during the time period between NACRT rounds (**Table 2** and **Figure 2**).

**Table 2:** Differences between CR and PR for texture parameters.

Quantitative parameters	CR (n=16) *x̅* ± *S*	PR (n=16)* x̅* ± *S*	P Value
Energy			
1^st^ NACRT	0.027 ± 0.010	0.028 ± 0.009	0.669
Change	−0.007 ± 0.013	−0.008 ± 0.009	1.000
2^nd^ NACRT	0.020 ± 0.007	0.020 ± 0.005	0.809
Entropy			
1^st^ NACRT	5.775 ± 0.440	5.779 ± 0.348	0.985
Change	0.309 ± 0.641	0.998 ± 0.803	0.032^**^
2^nd^ NACRT	6.084 ± 0.442	6.777 ± 0.827	0.047^**^
Inertia			
1^st^ NACRT	5.542 ± 5.871	4.488 ± 2.864	0.867
Change	3.484 ± 5.917	2.680 ± 2.866	0.491
2^nd^ NACRT	9.027 ± 5.122	7.169 ± 3.525	0.381
Correlation			
1^st^ NACRT	0.127 ± 0.057	0.130 ± 0.041	0.696
Change	−0.054 ± 0.071	−0.046 ± 0.037	0.590
2^nd^ NACRT	0.073 ± 0.051	0.083 ± 0.040	0.341
Cluster shade			
1^st^ NACRT	3.783 ± 21.441	1.341 ± 17.837	1.000
Change	−25.878 ± 48.778	−16.604 ± 25.707	0.590
2^nd^ NACRT	−22.095 ± 41.193	−15.263 ± 27.953	0.491
Cluster prominence			
1^st^ NACRT	941.802 ± 701.885	736.507 ± 463.486	0.445
Change	626.211 ± 1063.668	386.524 ± 783.817	0.724
2^nd^ NACRT	1568.012 ± 846.041	1123.031 ± 650.653	0.080
IDM			
1^st^ NACRT	0.468 ± 0.092	0.482 ± 0.067	0.590
Change	−0.890 ± 0.121	−0.080±−0.059	0.515
2^nd^ NACRT	0.378 ± 0.095	0.402 ± 0.079	0.402

** means significant difference.

### Diagnostic Performance of Vascular Permeability and Texture Parameters Between the CR and PR Groups

**Figure 3:**
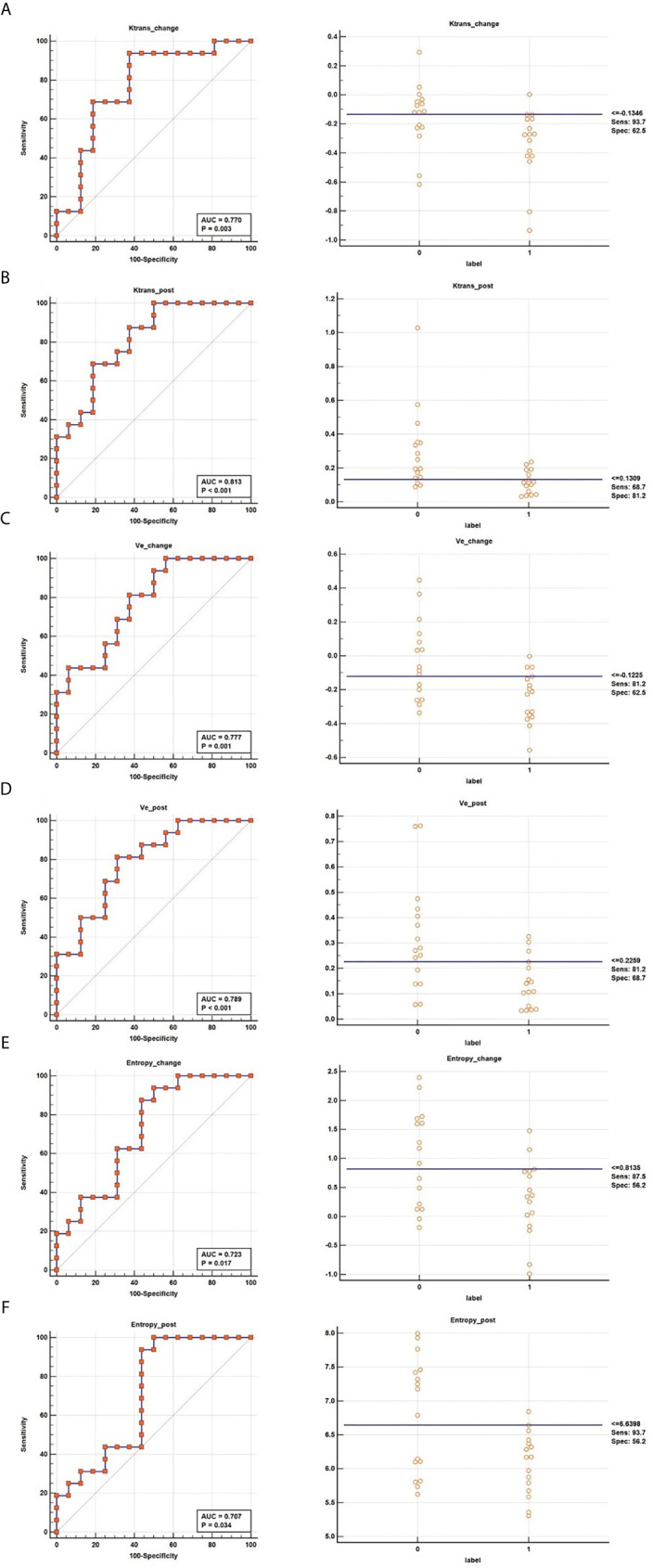
ROC curves of Ktrans_change_ **(A)**, Ktrans_post_ **(B)**, ve_change_ **(C)**, ve_post_ **(D)**, entropy_change_ **(E)**, and entropy_post_ **(F)** for determining the response to NACRT.

Six parameters of post-treatment Ktrans, ve, entropy, and the changes in these parameters showed good diagnostic ability (AUC>0.7) for differentiating between the CR and PR groups, namely, Ktrans_post_ (Ktrans after the second NACRT, AUC=0.813), Ktrans _change_ (Ktrans in change, AUC=0.770), ve_change_ (AUC=0.777), ve_post_ (AUC=0.789), entropy_change_ (AUC=0.723), and entropy_post_ (AUC=0.707) (**Table 3** and **Figure 3**).

**Table 3:** Diagnostic ability of vascular permeability parameters and texture parameters according to response groups.

Quantitative parameters	AUC	95% CI	Cutoff value	Sensibility (%)	Specificity (%)
Ktrans_change_	0.770	0.587–0.899	−0.1346	93.7	62.5
Ktrans_post_	0.813	0.636–0.928	0.1309	68.7	81.2
ve_change_	0.777	0.596–0.905	−0.1225	81.2	62.5
ve_post_	0.789	0.609–0.913	0.2259	81.2	68.7
entropy_change_	0.723	0.537–0.865	0.8135	87.5	56.2
entropy_post_	0.707	0.520–0.854	6.6398	97.7	56.2

### Selection of Vascular Permeability and Texture Parameters and Building a Combined Model

**Figure 4:**
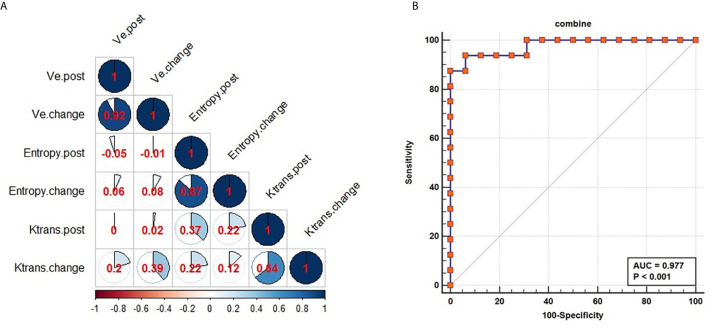
Performance of combined parameters for diagnosis in NACRT. Correlation coefficients for all parameters **(A)**; the AUC value for the combination of the parameters was 0.977 **(B)**.

P-values associated with Ktrans _post_, Ktrans _change_, ve_post_, ve_change_, entropy_post_ and entropy__change_ were of <0.05. After excluding factors associated with Spearman correlation coefficients of > 0.6, we retained Ktrans __post,_ve__post_ and entropy__post_ parameters, which wereKep included in a logistic regression model named Model_post_ predicting NACRT response (**Table 4** and **Figure 4A**), expressed as follows:

Equation 4 $$f(x)=\frac{1}{1+e^{-(26.772+Ktrans_{post}\times -17.010+Ve_{post}\times -15.854+Entropy_{post}\times -3.235)}}$$

**Table 4:** Performance of a logistic regression model with a combination of Ktrans, Ve and Entropy.

Quantitative Parameters	Coefficients	Std Error	Wald	P Value	OR	95% CI
Ktranspost	−17.010	8.737	3.790	0.05	0.000	1.50E-15–1.121
ve_post_	−15.854	7.869	4.060	0.044	0.000	2.61E-14–0.6495
entropy_post_	−3.235	1.605	4.061	0.044	0.039	0.0017–0.9153
intercept	26.772	11.699	5.237	0.0221		

The AUC of the model suggested excellent diagnostic ability (AUC=0.977, **Figure 4B**). The combined model was able to differentiate the CR from PR groups. At same time, the combined model of Model_change_ and Model_change-post_ were built (**Supplementary Equations 8**, **9** in **Supplementary Material 1**). Delong test was used to compare diagnostic performance among the three models, revealing no significant differences (Model_change_ vs Model_post_: P=0.1263, Model_change_ vs Model_change-post_: P=0.099, Model_post_ vs Model_change-post_: P=0.479, **Supplementary Table 1** and **Supplementary Figure 1** in **Supplementary Material 1**). As Model_post_ was the least complex and most straightforward to use in quantitative analysis, it was used to evaluate the response to neoadjuvant chemoradiotherapy in patients with esophageal squamous cell carcinoma.

## Discussion

This study demonstrated that the vascular permeability and texture parameters obtained from DCE-MRI scans can be used to evaluate tumor response after NACRT. Furthermore, the combination of vascular permeability parameters with texture parameters can be used to build a model that assesses tumor responses between the CR and PR groups. In this study, six post-treatment parameters (Ktrans, ve, entropy, and changes in these parameters) were significantly different between the CR and PR groups, showing good diagnostic ability for differentiating between the groups. The combined model showed a significant diagnostic ability, with the associated AUC value higher than the AUC values associated with each of the parameters separately.

Jinrong et al. found that vascular permeability parameters can be used to assess the response in patients receiving neoadjuvant chemotherapy (18). The Ktrans (transfer constant) was characterized as the diffusive transport of the Gd-DTPA-BMA contrast across the vascular endothelium (19), which suggests that Ktrans value is proportional to vascular permeability. In this study, the vascular permeability parameters at different time points (before the first NACRT and after the second NACRT) were used to predict tumor response. Dijkhoff used DCE-MRI scanning after chemoradiotherapy to evaluate tumor responses (20), similar to the studies by Jinrong (19). The previous studies also did not observe any significant differences in the vascular permeability parameters between the PR and CR groups before NACRT. After the second NACRT, this study found that the Ktrans of patients in the CR group was lower than that in the PR group, indicating that the number of blood vessels or vascular permeability value were lower in the former than in the latter group. Hironaka demonstrated that tumors with CR have a downregulated expression of CD31 and vascular endothelial growth factor (21). The ve value based on the DCE-MRI scans reflects the ratio of the volume of the contrast agent in the extravascular extracorporeal space, which is indicative of tumor proliferation. Tuillie et al. have shown that when tumor grade increases, pathological tumor volume and cell density value also increase (22); meanwhile, Chen et al. demonstrated that a higher ve value is associated with a higher tumor T stage (23). In the present study, the rate of tumor proliferation in the PR group was higher than that in the CR group; thus, the value of ve in the PR group was higher than that in the CR group. Even though Kep and vp values in the CR group were lower than those in the PR group, no significant differences were observed; this finding was not consistent with that reported by Jinrong (19). Han showed that texture features could be examined using diffusion-weighted imaging and that they could serve as useful biomarkers in the prognostication of patients with ESCC after chemoradiotherapy (24). The present study has shown that entropy can be used as a texture marker to distinguish between the PR and CR groups. Entropy measures the randomness of the distribution of values of the coefficients over various intensity levels. If the value of entropy is high, then the distribution has more intensity levels in the image. Entropy definitions are presented in **Supplementary Material 1**. It has been reported that the analysis of texture using DCE-MRI scanning can help identify tumor types, for example, breast cancer subtypes (25), and the histology grade in clear cell renal cell carcinoma (26). No previous prospective study has combined DCE-MRI scanning with texture analysis to predict NACRT response in patients with ESCC. The present ROC curve analysis of Ktrans, ve, and entropy revealed AUC of >0.7, which was satisfactory. The logistic regression model using Ktrans _post, ve_post, and entropy parameters was able to predict the response to NACRT, yielding AUC values higher than those associated with each parameter separately (Ktrans, Kep and ADC). Intra-tumoral heterogeneity is used to evaluate the degree of tumor aggressiveness, and it is an important imaging biomarker to predict tumor prognosis (27). Furthermore, tumor vascular normalization to moderate the hypoxia in the tumor can also be considered as a response to therapy; finally, vascular morphology and permeability parameters are among the gold standards for evaluating tumor vascular normalization (28). Therefore, intra-tumoral biomarkers (texture parameters) and biomarkers of vascular permeability (vascular permeability parameters) were combined to build a multivariable model (AUC=0.977), which could improve the degree of diagnostic ability in predicting PR and CR. This study provides a non-invasive method that is more comprehensive that a single index parameter.

There are several limitations to this study. First, the sample size was small, which may have biased the presented estimates. Second, we extracted seven texture parameters that are useful in a research context. However, more texture parameters can be extracted from MRI and other imaging modalities, which involve more sequences; in fact, the gold standard for texture parameters remains to be established. Normalization of texture parameters is another critical issue. Third, we did not differentiate the molecular types of ESCC. Therefore, further studies using large sample sizes are required to predict the response of different molecular types of ESCC to NACRT using DCE-MRI scanning combined with texture analysis.

In conclusion, the texture and vascular permeability parameters extracted from the DCE-MRI scans showed significant differences between the PR and CR groups. These parameters can be used as biomarkers to assess the response to NACRT. The use of a model that combines vascular permeability and texture parameters can improve prognostication after NACRT in patients with ESCC.

## Data Availability Statement

The raw data supporting the conclusions of this article will be made available by the authors, without undue reservation.

## Ethics Statement

The studies involving human participants were reviewed and approved by the ethics committee of Zhejiang Taizhou Hospital. The patients/participants provided their written informed consent to participate in this study.

## Author Contributions

Guarantor of integrity of entire study, WW and WJ. Study concepts/study design or data acquisition of data analysis/interpretation, all authors. Manuscript drafting or manuscript revision for important intellectual content, all authors. Manuscript final version approval, all authors. Agrees to ensure any questions related to the work are appropriately resolved, all authors. Literature research, WJ, JW, RZ, and MW. Clinical studies, WJ, JW, and WW. Statistical analysis, MK and PP. Manuscript editing, WJ, PP, and WW. All authors contributed to the article and approved the submitted version.

## Conflict of Interest

The authors declare that the research was conducted in the absence of any commercial or financial relationships that could be construed as a potential conflict of interest.

## References

[1] Bray F Ferlay J Soerjomataram I Siegel RL Torre LA Jemal A. Global Cancer Statistics 2018: GLOBOCAN Estimates of Incidence and Mortality Worldwide for 36 Cancers in 185 Countries. CA Cancer J Clin (2018) 68:394–424. 10.3322/caac.21492 30207593

[2] Shuhei M Tomoyuki I Hirofumi K Yuko K. Neoadjuvant Treatment Strategy for Locally Advanced Thoracic Esophageal Cancer. Ann Gastroenterol Surg (2019) 3:269–75. 10.1002/ags3.12243 PMC6524122 31131355

[3] Mao Y Gao S Wang Q Shi X Li Y He J. Epidemiological Characteristic and Current Status of Surgical Treatment for Esophageal Cancer by Analysis of National Registry Database. Chin J Oncol (2020) 42:228–33. 10.3760/cma.j.cn112152-20191112-00729 32252202

[4] Burmeister B Smithers BM Gebski V. Surgery Alone Versus Chemoradiotherapy Followed by Surgery for Resectable Cancer of the Oesophagus: A Randomized Controlled Phase III Trial. Lancet Oncol (2005) 6:659–68. 10.1016/S1470-2045(05)70288-6 16129366

[5] Sjoquist KM Burmeister BH Smithers BM. Survival After Neoadjuvant Chemotherapy or Chemoradiotherapy for Resectable Oesophageal Carcinoma: An Updated Meta-Analysis. Lancet Oncol (2011) 12:681–92. 10.1016/S1470-2045(11)70142-5 21684205

[6] Shpiro J Van Lanschot JJB Hulshof M van Hagen P van Berge Henegouwen MI Wijnhoven BPL. Neoadjuvant Chemoradiotherapy Plus Surgery Versus Surgery Alone for Oesophageal or Junctional Cancer (CROSS): Long-Tern Results of a Randomised Controlled Trial. Lancet Oncol (2015) 16:1090–8. 10.1016/S1470-2045(15)00040-6 26254683

[7] Yang H liu H Chen Y. Neoadjuvant Chemoradiotherapy Followed by Surgery Versus Surgery Alone for Locally Advanced Squamous Cell Carcinoma of the Esophagus (NEOCRTEC5010): A Phase III Multicenter, Randomized, Open-Label Clinical Trial. J Clin Oncol (2018) 36:2796–803. 10.1200/JCO.2018.79.1483 PMC6145832 30089078

[8] Soror T Koh G Zhao KL Ismail M Badakhshi H. Impact of Pathological Complete Response Following Neoadjuvant Chemoradiotherapy in Esophageal Cancer. J Thorac Dis (2018) 10:4069–76. 10.21037/jtd.2018.06.85 PMC6106005 30174850

[9] Qiu B Wang D Yang H Xie WH Liang Y Peiqiang C. Combined Modalities of Magnetic Resonance Imaging, Endoscopy and Computed Tomography in the Evaluation of Tumor Responses to Definitive Chemoradiotherapy in Esophageal Squamous Cell Carcinoma. Radiother Oncol (2016) 121:239–45. 10.1016/j.radonc.2016.09.017 27793445

[10] Gollub MJ Tong T Weiser M Zheng JT Gonen M Zakian KL. Limited Accuracy of DCE-MRI in Identification of Pathological Complete Responders After Chemoradiotherapy Treatment for Rectal Cancer. Eur Radiol (2017) 27:1605–12. 10.1007/s00330-016-4493-1 PMC5570543 27436029

[11] Oberholzer K Phohlmann A Schreiber W Midenberger P Kunz P Schmidberger H. Assessment of Tumor Microcirculation With Dynamic Contrast-Enhanced MRI in Patients With Esophageal Cancer: Initial Experience. J Magn Reason Imaging (2008) 27:1296–301. 10.1002/jmri.21305 18504749

[12] Li Z Han C Wang L Zhu J Yin y Li B. Prognostic Value of Texture Analysis Based on Pretreatment Dwi-Weighted MRI for Esophageal Squamous Cell Carcinoma Patients Treated With Concurrent Chemo-Radiotherapy. Front Oncol (2019) 9:1057. 10.3389/fonc.2019.01057 31681593 PMC6811607

[13] Qu J Shen C Qin J Wang Z Liu Z Guo J. The MR Radiomic Signature can Predict Preoperative Lymph Node Metastasis in Patients With Esophageal Cancer. Eur Radiol (2018) 29:906–14. 10.1007/s00330-018-5583-z 30039220

[14] Mandard AM Dalibard F Mandard JC Henry AM Petiot JF Roussel A. Pathologic Assessment of Tumor Regression After Preoperative Chemoradiotherapy of Esophageal Carcinoma. Clinicopathologic Correlations. Cancer (1994) 73:2680–6. 10.1002/1097-0142(19940601)73:11<2680::AID-CNCR2820731105>3.0.CO;2-C 8194005

[15] Lei H Yun FZ Wang L Liang L Dong X Xue SL. Quantitative Evaluation of Vertebral Microvascular Permeability and Fat Fraction in Alloxan-Induced Diabetic Rabbits. Radiology (2018) 287:128–36. 10.1148/radiol.2017170760 29156149

[16] Wake N Chandarana H Rusinek H Fujimoto K Moy L Sodickson DK. Accuracy and Precision of Quantitative DCE-MRI Parameters: How Should One Estimate Contrast Concentration? Magn Reson Imaging (2018) 52:16–23. 10.1016/j.mri.2018.05.007 29777820 PMC6102067

[17] Khalifa F Soliman A EI-Baz A El-Ghar MA El-Diasty T Gimel’farb G. Model and Methods for Analyzing DCE-MRI: A Review. Med Phys (2014) 41:124301. 10.1118/1.4898202 25471985

[18] Yanan L Ling M Jinrong Q Jianjun Q Zhaoqi W Jia G. The Value of GRASP on DCE-MRI for Assessing Response to Neoadjuvant Chemotherapy in Patients With Esophageal Cancer. BMC Cancer (2019) 19:999. 10.1186/s12885-019-6247-3 31651280 PMC6814031

[19] Tofts PS Brix G Buckley DL Evelhoch JL Henderson E Knopp MV. Estimating Kinetic Parameters From Dynamic Contrast-Enhanced T1-weighted MRI of a Diffusible Tracer: Standardized Quantities and Symbols. J Magn Reason Imaging (1999) 10:223–32. 10.1002/(SICI)1522-2586(199909)10:3<223::AID-JMRI2>3.0.CO;2-S 10508281

[20] Dijkhoff R Beets-Tan R Larbregts DMJ Beets GL Mass M. Value of DCE-MRI for Staging and Response Evaluation in Rectal Cancer: A Systematic Review. Eur J Radiol (2017) 95:155–68. 10.1016/j.ejrad.2017.08.009 28987662

[21] Hironaka S Hasebe T Ochiai A Boku N Yoshida S Saitoh H. Biopsy Specimen Microvessel Density is a Useful Prognostic Marker in Patients With T(2-4)M(0) Esophageal Cancer Treated With Chemoradiotherapy. Clin Cancer Res (2002) 8:124–30. 10.1016/S0531-5131(01)00620-3 11801548

[22] Tullie LG Sohn HM Zylstra J Mattsson F Griffin N Sharma N. A Role for Tumor Volume Assessment in Resectable Esophageal Cancer. Ann Surg Oncol (2016) 23(9):3063–70. 10.1245/s10434-016-5228-x 27112584

[23] Chen YL Li R Chen TW Ou J Zhang XM Chen F. Whole-Tumor Histogram Analysis of Pharmacokinetic Parameters From Dynamic Contrast-Enhanced MRI in Resectable Oesophageal Squamous Cell Carcinoma can Predict T-stage and Regional Lymph Node Metastasis. Eur J Radiol (2019) 112:112–20. 10.1016/j.ejrad.2019.01.012 30777199

[24] Li B Li Z Han C. Prognostic Value of Pretreatment Diffusion Weighted Magnetic Resonance Imaging Based Texture in Concurrent Chemo-Radiotherapy of Esophageal Squamous Cell Cancer. Ann Oncol (2018) V29(Suppl 8N):viii214. 10.1093/annonc/mdy282.022

[25] Wang H Hu Y Li H Xie Y Wang X Wan W. Preliminary Study on Identification of Estrogen Receptor Positive Breast Cancer Subtypes Based on Dynamic Contrast-Enhanced Magnetic Resonance Imaging (DCE-MRI) Texture Analysis. Gland Surg (2020) 9:622–8. 10.21037/gs.2020.04.01 PMC7347815 32775251

[26] Dwivedi DK Xi Y Kapur P Madhuranthakanm AJ Lewis MA Pedrosa I. Magnetic Resonance Imaging Radiomics Analysis for Prediction of High-Grade Histology and Necrosis in Clear Cell Renal Cell Carcinoma: Preliminary Experience. Clin Genitourin Cancer (2020) S1558-7673:30119–1. 10.1016/j.clgc.2020.05.011 PMC7680717 32669212

[27] Wong CK Chan SC Ng SH Hsieh CH Cheng NM Liao CT. Textural Features on 18F-FDG PET/CT and Dynamic Contrast-Enhanced MR Imaging for Predicting Treatment Response and Survival of Patients With Hypopharyngeal Carcinoma. Med (Baltimore) (2020) 98:e16608. 10.1097/MD.0000000000016608 PMC6831375 31415354

[28] Li W Quan YY Li Y Lu L Cui M. Monitoring of Tumor Vascular Normalization: The Key Points From Basic Research to Clinical Application. Cancer Manag Res (2018) 10:4163–72. 10.2147/CMAR.S174712 PMC6175544 30323672

