# Supplementary material for: Diagnostic Performance of Vascular Permeability and Texture Parameters for Evaluating the Response to Neoadjuvant Chemoradiotherapy in Patients With Esophageal Squamous Cell Carcinoma
Source: Front Oncol. 2021 May 18;11:604480. doi: 10.3389/fonc.2021.604480 (PMC8168434; doi:10.3389/fonc.2021.604480)
Supplement: Supplementary file 1 [file DataSheet_1.pdf]

Supplementary materials 1.

Texture parameters Description (1)

1.1 Energy

This feature returns the sum of squared elements in the GLCM. Range=[0 1]. Energy is 1 for a constant image. Is high when image has very good homogeneity or when pixels are very similar The property Energy is also known as uniformity, uniformity of energy, and angular second moment.

$$\text{Energy} = \sum_{i,j} g(\text{i, j})^2 \qquad \text{Equation 1}$$

*g is a GLCM

Where i,j are the spatial coordinates of g (i,j)

1.2 Entropy

The entropy measures the randomness of the distribution of the coefficients values over the intensity levels. If the value of entropy is high, then the distribution is among more intensity levels in the image. This measurement is the inverse of energy. A simple image has low entropy while a complex image has high entropy. Entropy can be defined as:

$$Entropy = -\sum_{i=1}^{Ni} p(i) log_2 p(i) \qquad \text{Equation 2}$$

1.3 Inertia

It reflects the clarity of the image and texture groove depth. The contrast is proportional to the texture groove, high values of the groove produces more clarity, in contrast small values of the groove will result in small contrast and fuzzy image.

$$Inertia = \sum_{i,j} (\text{i} - \text{j})^2 g(i,j) \qquad \text{Equation 3}$$

1.4 Correlation

Correlation measures the linear dependency of grey levels of neighboring pixels, in other words, it measures the similarity of the grey levels in neighboring pixels, tells how correlated a pixel is to its neighbor over the whole image.

$$Correlation = -\sum_{n=1} \left(\frac{(i-\mu)(j-\mu)g(i,j)}{\sigma^2}\right) \qquad \text{Equation 4}$$

1.5 Clustershade

Cluster analysis or clustering is the task of grouping a set of objects in such a way that objects in the same group (cluster) are more similar (in some sense or another) to each other than to those in other groups (clusters). It is a common technique for statistical data analysis.

$$Clustershade = \sum_{i,j} ((\text{i} - \mu) + (j - \mu))^3 g(i,j) \qquad \text{Equation 5}$$

1.6 Cluster prominence

Cluster prominence is a measure of asymmetry of a given distribution, high values of this feature indicate that the symmetry of the image is low, in medical imaging low values of cluster prominence represent a smaller peak for the image grey level value and usually the grey level difference between the forms is small.

$$Cluster\ prominence = \sum_{i,j} \left((\text{i} - \mu) + (\text{j} - \mu)\right)^4 g(i,j) \qquad \text{Equation 6}$$

1.7 IDM (Inverse Difference Moment)

Inverse Difference Moment (IDM) is the local homogeneity. It is high when local gray level is uniform and inverse GLCM is high. IDM weight value is the inverse of the contrast weight.

$$IDM = \sum \sum \frac{1}{1+(i-j)^2} g(i,j)$$ Equation 7

Reference
1. P. Mohanaiah, P. Sathyanarayana and L. GuruKumar, Image texture feature extraction using GLCM approach, International Journal of Scientific and Research Publications, Volume 3, Issue 5, May 2013.

Diagnostic performance of $\text{Model}_{\text{change}}$ and $\text{Model}_{\text{change-post}}$ (2)

The combined model was built $\text{Model}_{\text{change}}$ based on the changes to the three parameters (Ktranschange, vechange and entropychange). The equation representing $\text{Model}_{\text{change}}$ built by the changes of Ktrans, ve, and entropy is:

$$f(x) = \frac{1}{1+e^{-(-0.628 - Ktrans_{change} \times 3.449 - Ve_{change} \times 6.943 - entropy_{change} \times 1.764)}}$$ Equation 8

$\text{Model}_{\text{change-post}}$ based on the changes and post-treatment values of Ktrans, ve and entropy was expressed as:

$$f(x) = \frac{1}{1+e^{-(21.229 - Ktrans_{post} \times 35.709 + Ktrans_{change} \times 6.275 - Ve_{post} \times 12.316 - Ve_{change} \times 13.081 - entropy_{post} \times 1.611 - entropy_{change} \times 3.811)}}$$ Equation 9

Table 1. Diagnostic performance of $\text{Model}_{\text{change}}$, $\text{Model}_{\text{post}}$ and $\text{Model}_{\text{change-post}}$

	AUC	95% CI		Youden index	Sensitivity	Specificity
		Upper	Lower			
$\text{Model}_{\text{change}}$	0.895	0.975	0.735	0.687	0.875	0.812
$\text{Model}_{\text{post}}$	0.977	1.000	0.850	0.875	0.937	0.937
$\text{Model}_{\text{change-post}}$	0.980	1.000	0.857	0.875	0.938	0.938

$\text{Model}_{\text{change}}$ is based on the changes to Ktrans, ve, and entropy values (Equation 1). $\text{Model}_{\text{post}}$ is based on post-treatment values of Ktrans, ve, and entropy (Equation 3 in the main text). $\text{Model}_{\text{change-post}}$ is based on the changes to and post-treatment values of Ktrans, ve, and entropy (Equation 2).

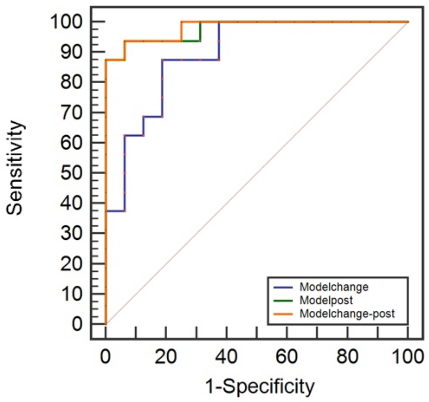

Figure 1. Comparison of diagnostic performance among $\text{Model}_{\text{change}}$, $\text{Model}_{\text{post}}$, and $\text{Model}_{\text{change-post}}$
